# Neural-based modeling adsorption capacity of metal organic framework materials with application in wastewater treatment

**DOI:** 10.1038/s41598-022-08171-7

**Published:** 2022-03-08

**Authors:** Mozhgan Parsaei, Elham Roudbari, Farhad Piri, A. S. El-Shafay, Chia-Hung Su, Hoang Chinh Nguyen, May Alashwal, Sami Ghazali, Mohammed Algarni

**Affiliations:** 1 School of Chemistry, College of Science, University of Tehran, Tehran, Iran; 2 Department of Chemistry, Faculty of Science, Shahid Bahonar University of Kerman, Kerman, Iran; 3 Electrical Engineering Department, Amirkabir University of Technology, Hafez Avenue, Tehran, Iran; 4 Department of Mechanical Engineering, College of Engineering, Prince Sattam Bin Abdulaziz University, Alkharj, 11942 Saudi Arabia; 5 Department of Chemical Engineering, Ming Chi University of Technology, New Taipei City, Taiwan; 6 Faculty of Applied Sciences, Ton Duc Thang University, Ho Chi Minh City, 700000 Vietnam; 7 Department of Computer Science, Jeddah International College, Jeddah, Saudi Arabia; 8 Mechanical and Materials Engineering Department, Faculty of Engineering, University of Jeddah, P.O. Box 80327, Jeddah, 21589 Saudi Arabia; 9 Mechanical Engineering Department, Faculty of Engineering, King Abdulaziz University, P.O. Box 344, Rabigh, 21911 Saudi Arabia

**Keywords:** Chemical engineering, Environmental impact

## Abstract

We developed a computational-based model for simulating adsorption capacity of a novel layered double hydroxide (LDH) and metal organic framework (MOF) nanocomposite in separation of ions including Pb(II) and Cd(II) from aqueous solutions. The simulated adsorbent was a composite of UiO-66-(Zr)-(COOH)_2_ MOF grown onto the surface of functionalized Ni_50_-Co_50_-LDH sheets. This novel adsorbent showed high surface area for adsorption capacity, and was chosen to develop the model for study of ions removal using this adsorbent. A number of measured data was collected and used in the simulations via the artificial intelligence technique. Artificial neural network (ANN) technique was used for simulation of the data in which ion type and initial concentration of the ions in the feed was selected as the input variables to the neural network. The neural network was trained using the input data for simulation of the adsorption capacity. Two hidden layers with activation functions in form of linear and non-linear were designed for the construction of artificial neural network. The model’s training and validation revealed high accuracy with statistical parameters of R^2^ equal to 0.99 for the fitting data. The trained ANN modeling showed that increasing the initial content of Pb(II) and Cd(II) ions led to a significant increment in the adsorption capacity (Qe) and Cd(II) had higher adsorption due to its strong interaction with the adsorbent surface. The neural model indicated superior predictive capability in simulation of the obtained data for removal of Pb(II) and Cd(II) from an aqueous solution.

## Introduction

Artificial intelligence (AI) has recently attracted much attention for simulating physical, biochemical, and chemical processes to provide a simulation tool for process understanding, optimization, and improvement. Basically, mathematical models and simulations are performed to understand the process, and minimize the operational costs, while maximizing the process efficiency^1–7^. Simulation and optimization of processes have been used in many different areas in the recent years^8–11^. The models developed using artificial intelligence techniques require measured data for training the algorithm. Also, selection of artificial intelligence algorithm depends on the process, and the degree of complexity^12,13^. Artificial intelligence-based simulation methodology and models can be employed for the systems that are too complex to be formulated via mechanistic models. Therefore, the artificial intelligence models can help simulate complex processes for optimization and process improvement^11,14,15^.

Recently, artificial intelligence models have been combined with computational fluid dynamics techniques to improve the simulations and reduce the computational costs. In this hybrid modeling approach, the CFD simulation results are used as the input for artificial intelligence model to predict fluid flow, heat transfer, mass transfer, chemical reactions, and multiphase flow systems^16–31^. The artificial intelligence models have shown to be more accurate and faster than the mechanistic models in terms of computation which make them attractive for implementation in prediction of physical and chemical processes. The main disadvantage of these modes is that they need measured data for training the process, and these models are not of pure predictive nature^13^.

These artificial intelligence models can be applied for simulation of ion adsorption to the surface of nanoporous materials with high accuracy. Adsorption has indicated to be an efficient technique for wastewater treatment with low content of impurities^32–34^. Due to the complexity of nanoporous materials, mechanistic models are difficult to be developed and artificial intelligence-based models are preferred in this case. Adsorption using nanoporous materials and nanocomposite materials have been recently simulated via semi-empirical and empirical correlations^35–40^. Good agreement has been obtained for implementing empirical models for simulation of adsorption process, however these models show poor applicability in considering the effect of various parameters on adsorption capacity of the used adsorbent in the process. These models have been developed for mesoporous silica and nanocomposite materials in removal of organic materials and heavy metals from water^11,41–52^.

Development of artificial intelligence models for simulation of ion adsorption onto the surface of nanomaterials would be attractive for design of materials and optimization of the process^53^. For example, Ayaz and Khan^54^ performed a survey on the application of AI techniques on the modeling of heavy metal contaminants removal from wastewater using Levenberg–Marquardt (LM) and scale conjugate Gradient (SCG). Yaqub et al.^55^ used ANN and adaptive neuro-fuzzy inference system (ANFIS) to investigate the prediction of Cr(VI) adsorption on polymer inclusion membranes. The modeling results confirmed the high accuracy of these models, but the ANN results were more reliable than the outcomes obtained from ANFIS model. Usually, if the model is validated through comparing with experimental results, then the model can be used to map the adsorption process and find the optimum conditions^56,57^.

In order to propose and implement a high-performance model for prediction of adsorption process using hybrid materials with nanostructure, herein we demonstrate for the first time simulation of a novel Ni_50_Co_50_-LDH-COOH/UiO-66(Zr)-(COOH)_2_ nanocomposite (LDH/MOF) in separation of Pb(II) and Cd(II) solutes from water considering various conditions by development of an artificial intelligence-based model^58^. Different adsorbents had been used as effective adsorbents for pollutant removal from aqueous media such as MOFs, natural materials, and mesoporous silica^59–62^. In the recent years, metal organic framework (MOF) adsorbents have attracted so many attentions for this application due to their fantastic properties^63,64^. These models outperformed the traditional empirical correlations in fitting adsorption data such as the well-known Langmuir adsorption model^13^. The work is conducted by development of artificial neural network model considering ion type as well as initial ion concentrations as the inputs, while the adsorption capacity of the nanocomposite was considered as the only simulated output by the neural model. Training and cross-validation are performed to evaluate the accuracy of the neural model in description of the ion removal via the novel Ni_50_Co_50_-LDH-COOH/ UiO-66(Zr)-(COOH)_2_ nanocomposite.

## Materials and methods

The model is developed for simulation of ions removal using a novel nanocomposite made of functionalized Ni_50_Co_50_-LDH-COOH/UiO-66(Zr)-(COOH)_2_. Two ions are considered in this study including Pb(II) and Cd(II) for removal from water at different conditions by changing the ions concentration. The ions were considered at different initial concentrations between 0.5 and 250 mg/L. The data are collected from literature^58^, and this work is focused on the simulation of adsorption using an artificial intelligence model. We have also provided a little description about the measurements. According to this research^58^ as the needed materials for production of adsorbent were in analytical grade and used as received. Zirconium (IV) chloride (ZrCl4), Nickel(II) nitrate hexahydrate, Pyromellitic acid, Cobalt(II) nitrate hexahydrate, (3-Aminopropyl)triethoxysilane, and Cadmium nitrate tetrahydrate were obtained from Sigma-Aldrich. Also, ethanol, ethylene glycol, sodium hydroxide, acetone, and hydrochloric acid were obtained from Merck, as reported in Ref.^58^.

The nanocomposite of LDH/MOF which is considered in this study has indicated that this novel adsorbent can be utilized for the separation and capture of ions owing to its adsorption surface area in the interconnected nanoporous network. Therefore, the separation performance of the nanocomposite for the separation of Cd(II) and Pb(II) ions were evaluated. As reported in literature^58^, the adsorption experiments were carried out in conventional batch mode at temperature of $T$ = 25 ℃, by variations of initial ions concentration in the feed solution. The adsorption capacity of the adsorbent which is used as the only simulated output is estimated using^53,58^:

1 $${Q}_{\mathrm{e}}=\left({C}_{\mathrm{i}}-{C}_{\mathrm{e}}\right)\times \left(\frac{V}{W}\right),$$

where ${Q}_{\mathrm{e}}$ and ${C}_{\mathrm{e}}$ denote the equilibrium capacity of the adsorbent (mg g^–1^) and the ion concentration (mg g^–1^) at the equilibrium^58^.

## Modeling and simulation

**Figure 1:**
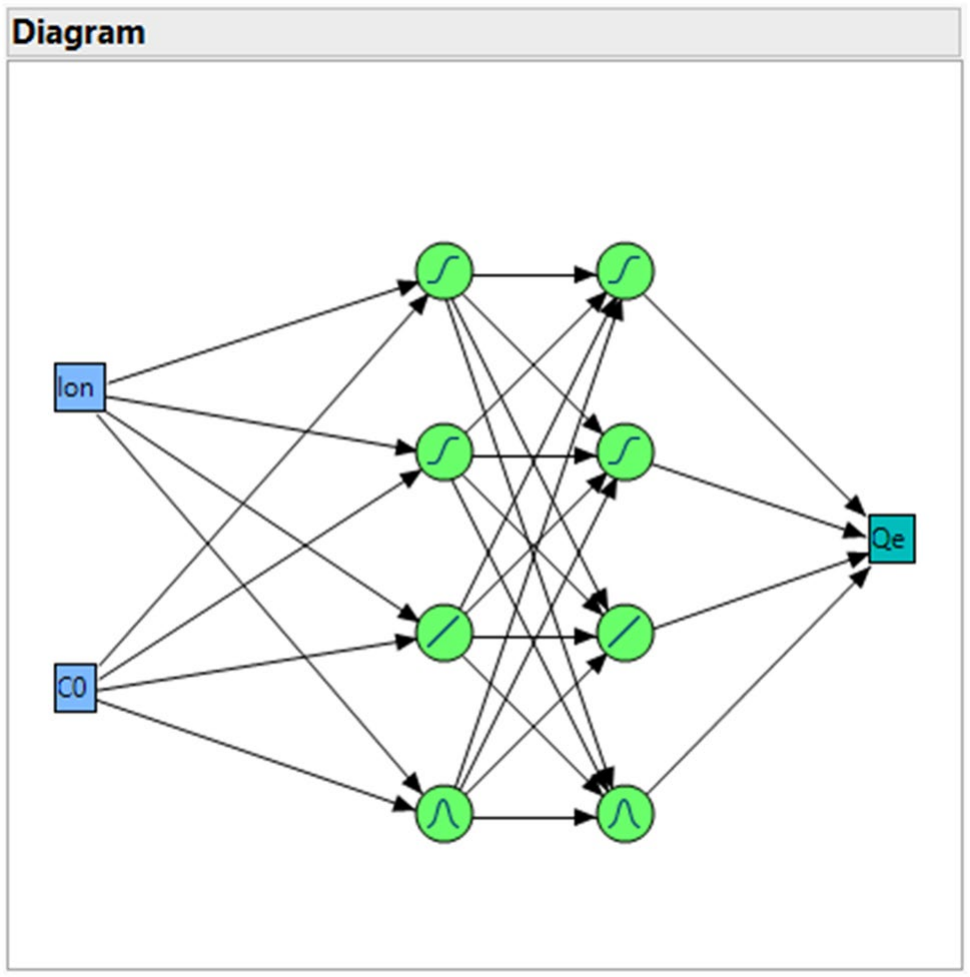
Design of the model employed in this work for simulation of adsorption.

Simulation of the adsorption data is here carried out using artificial neural network (ANN) method. In this technique, training the data is carried out to obtain the weight and bias parameters for the neural network^65–67^. The structure of the ANN model is represented in Fig. 1 in which the model has been developed by designing two hidden layers in which 2 non-linear (*TanH*), 1 linear, and 1 Gaussian function are used in the hidden layers’ nodes. The functions and nodes are utilized to estimate the output parameter which is adsorption capacity (Qe). The artificial neural network calculations were performed using *JMP* software which utilizes multi-layer perceptron neural network for simulation of the target value. The software is a powerful tool for prediction of output and making a relation between the input and output variables^68,69^. The simulations were performed using *KFold* as the validation technique in which *K* was set at 3 implying that the data points are split into three groups, and the best group with the minimum deviation is selected as the validation column. In this method of simulation using neural network, the designed hidden nodes are nonlinear functions of the original input variables.

## Results and discussion

### Simulation results

**Figure 2:**
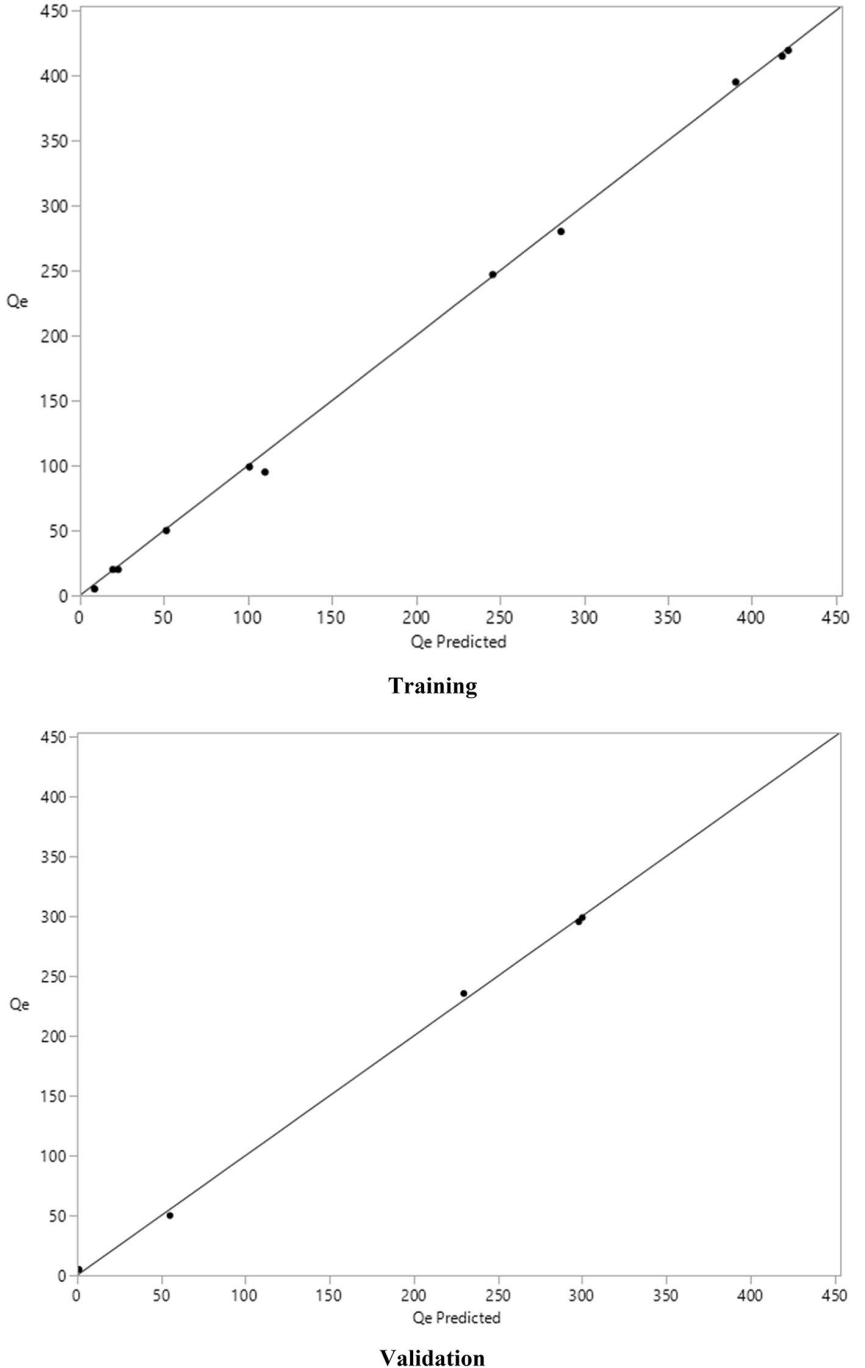
Training and validation data computed for removal of ions.

**Figure 3:**
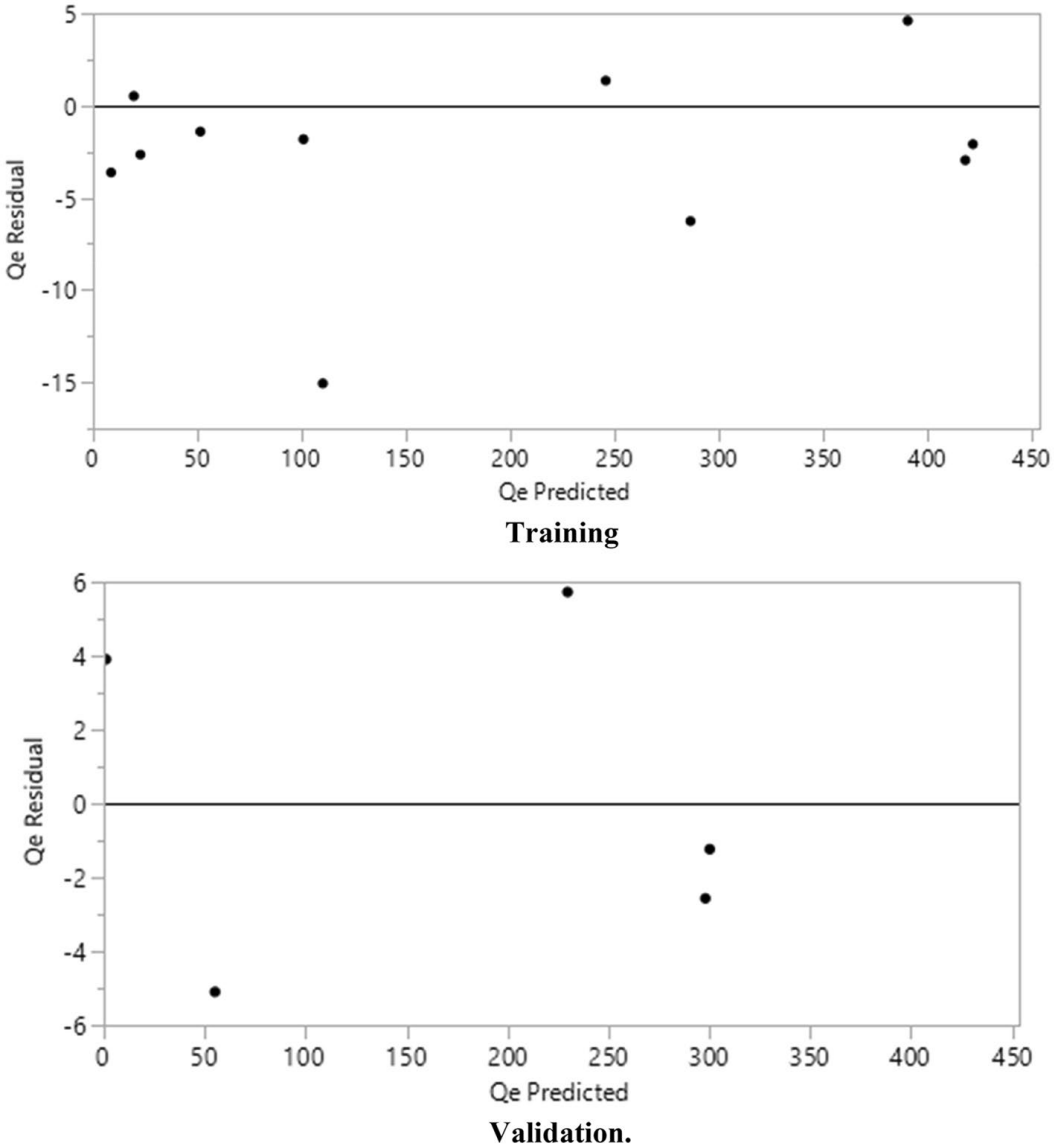
The residual of fitting.

The simulation results obtained by artificial neural network model are listed in Table 1 for the training and validation. Also, the predicted Qe values versus the experimental values are collected and compared in Fig. 2, while the residual of fittings are represented in Fig. 3. The results of simulated Qe using the neural network model confirmed that the great agreement has been achieved with high accuracy, and the R^2^ equal to 0.99 has been calculated for training and validation of the network in this work. Also other statistical parameters including SSE and RMSE indicated great values implying that the model has been properly trained^70^ and the model can simulate the process for removal of Pb(II) and Cd(II) from water by the nanocomposite adsorbent of functionalized LDH/MOF.

**Table 1:** Analysis of training/validation fitting.

Measures	Training	Validation
R^2^	0.9988514	0.9989469
RMSE	5.4591438	4.0546973
Mean Abs Dev	3.8497789	3.7071214
− LogLikelihood	34.278535	14.094073
SSE	327.82476	82.20285
Sum freq	11	5

**Figure 4:**
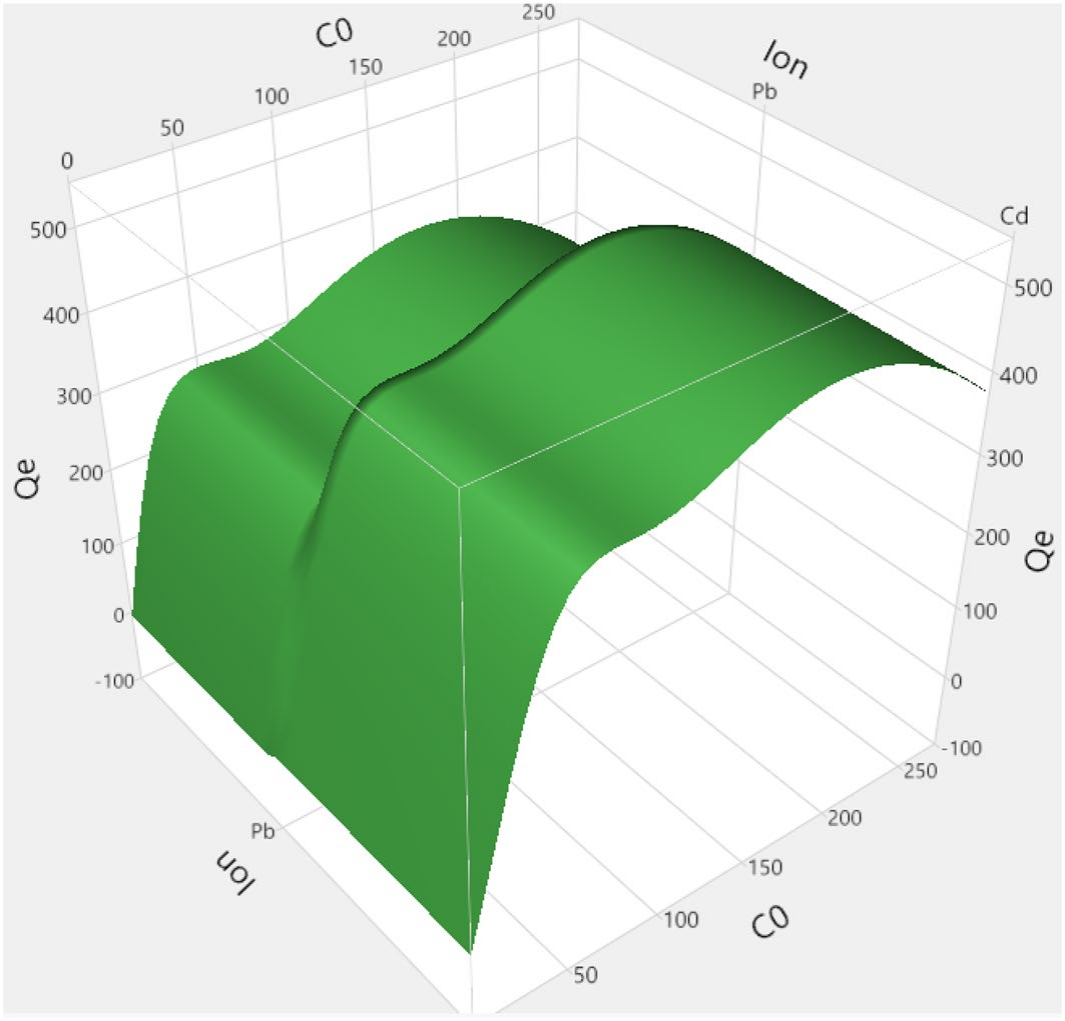
3D plot of predicted Qe.

**Figure 5:**
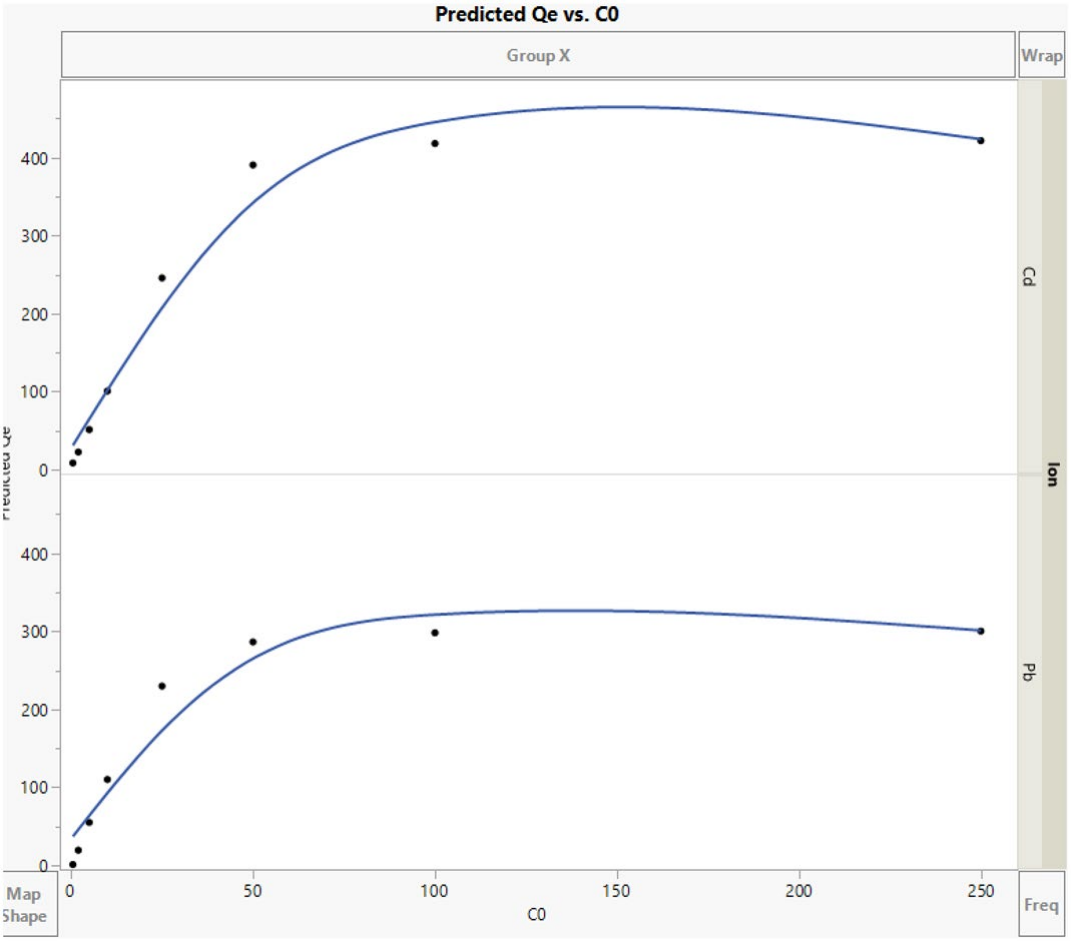
2D plot of predicted Qe.

**Figure 6:**
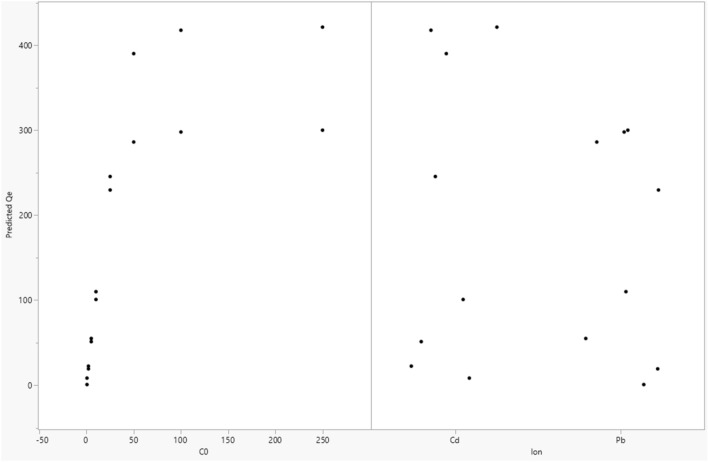
Scatterplot matrix of predicted Qe.

**Figure 7:**
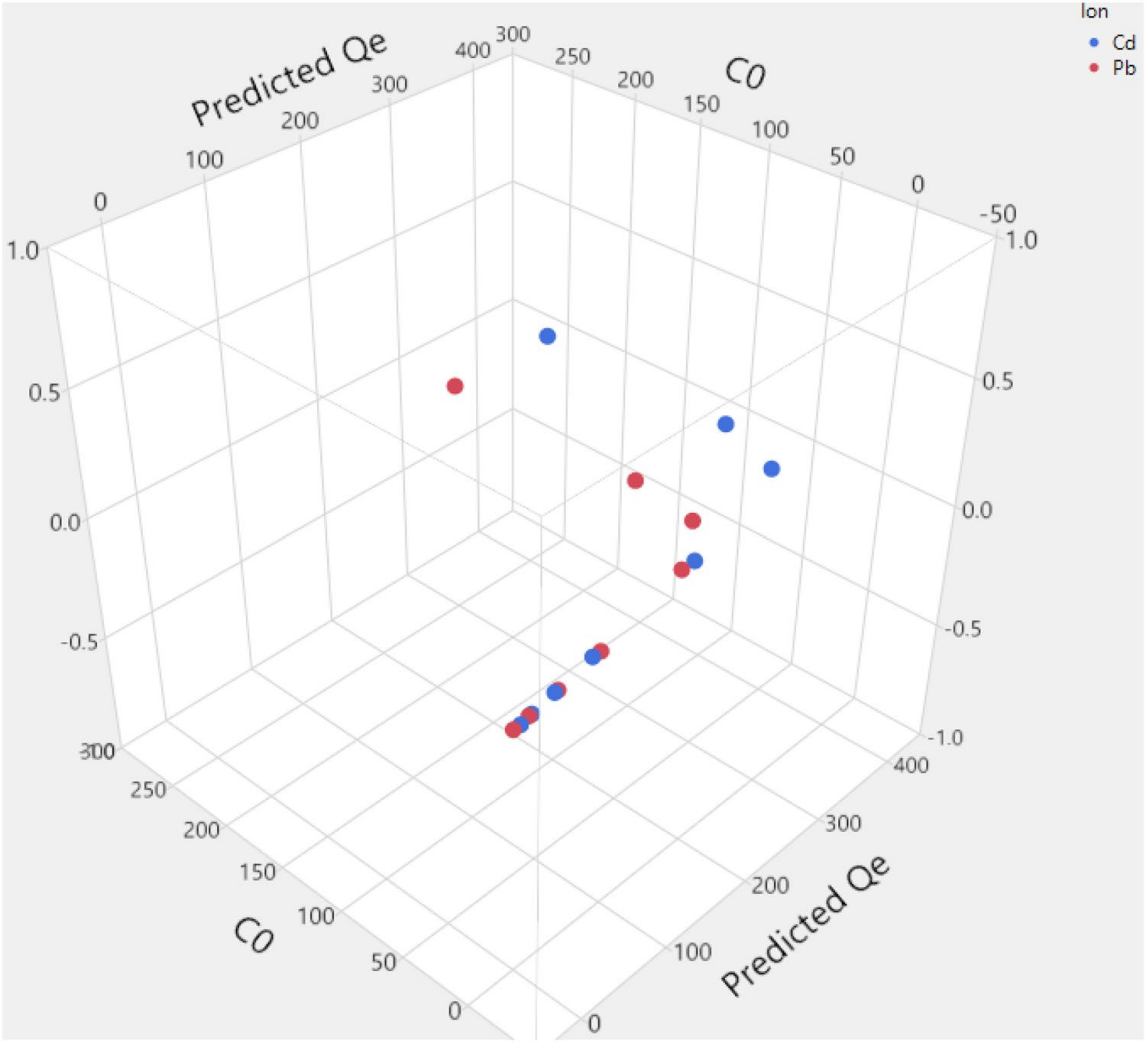
Scatterplot 3D of predicted Qe.

The fitted and trained model was further used to interpret the obtained adsorption data^71^. The results of simulations as 3D and 2D plots of Qe are illustrated in Figs. 4 and 5. Also, the scatterplots of predicted Qe using the developed ANN are illustrated in Figs. 6 and 7. In Fig. 4, the 3D plot of simulated Qe versus ion type and initial concentration of ions (C_0_) is indicated. It is observed that initial concentration can have effect on the adsorption capacity. The initial concentration can change the driving force for mass transfer of the solute from the bulk of feed solution towards the nanocomposite adsorbent. Indeed, increasing the initial content of the ions in the solution will increase the adsorption capacity (Qe) significantly as predicted by the developed neural network model. Moreover, the results presented in Fig. 5 indicate that Cd has higher adsorption on the surface of nanocomposite adsorbent which is due to its favorable interaction with the functional groups on the surface of adsorbent^53,58,70^. A review on different reported results for ML modeling methods and specially ANN method, for adsorption of Cd and Pb heavy metal ions from aquatic solution is reported in Table 2^72^.

**Table 2:** Comparison of different reports on ML modeling methods, especially ANN method, for Cd and Pb heavy metal ions removal^72^.

ML models	Metrics	Model input	Model output	Result of research	Ref.
ANN	RMSE, R^2^	Mass of the solid adsorbent, size of column, fluid velocity, bed size and concentration of ions	Separation efficiency	Adsorption of Cd heavy metal on immobilized Bacillus subtilis beads. ANN model showed excellent capability for removal efficiency prediction	^73^
LSSVR, ANN, GP, ANFISPSO, PNR	MSE, R^2^, AARD	Electronegativity, first ionization energy, initial pH and equilibrium pH	Ionic species sorbed	Adsorption of Zn, Ni, Cd, Pb heavy metal ions on natural zeolite. All the used models confirmed perfection in the simulation of adsorption process over testing and training trials	^74^
ANN	MSE, R^2^	Adsorbent weight, dye content, and ultrasonic	Adsorption capacity	Adsorption of Cd and Co on ZnO-NRs-AC. The obtained results showed that the ANN model were able to predict and model the adsorption process	^75^
ANN	RMSE, R^2^	Removal duration, C_0_, dosage	Separation efficiency	Adsorption of Pb(II) and Cu(II) ions on nanocomposites of rice straw and Fe_3_O_4_ nanoparticles. Proper prediction of adsorption process due to due to the minimum RMSE and maximum R-squared	^76^
ANN, ANFIS	MSE, R^2^	Straw pH, initial content of Cd(II), and biosorbent dose	Biosorption efficiency	Adsorption of Cd on rice. The proposed network by ANN capable in prediction of Cd adsorption with high accuracy	^77^
ANN	R^2^	Walnut shell-rice husk ratio, calcination duration and temperature	Sorption efficiency	Adsorption of Cd on Nano-magnetic walnut shell-rice husk	^78^
ANN, MLR	R^2^	Treatment time, adsorbent weight, C_0_, solution pH	Separation efficiency	Adsorption of Pb(II) on carboxylate-functionalized walnut shell (CFWS). Result confirmed ANN model was able to predict the Pb(II) removal more accurately compare to MLR	^79^
GA-ANN	MSE, R^2^	No. of adsorbent, solution pH, adsorbent weight, time, and initial content	Removal efficiency	Adsorption of Cd on natural waste materials (leaves of jackfruit, mango and rubber plants). The outcomes confirmed the accuracy of modelling and obtained results have good agreement with the experimental data	^80^
ANN	MSE, R^2^	C_0_, biosorbent weight, contact time	Removal efficiency	Biosorption of Cd, Pb, Ni by itaconic acid grafted poly (vinyl) alcohol encapsulated wok pulp. The developed ANN statistical model was successful in providing a valuable instrument	^81^
ANN	RMSE, R^2^ Mean Abs Dev − LogLikelihood SSE Sum Freq	Type and initial concentration of ions	Adsorption capacity	Adsorption of Pb(II) and Cd(II) ions on MOF/LDH nanocomposite. The high value of R^2^ and low value of RMSE confirmed the excellent performance of developed ANN model	This research

## Conclusions

Removal of Pb(II) and Cd(II) ions from water using a nanocomposite of MOF/LDH was studied in this work. Computational studies were carried out in order to simulate the process to understand the effect of parameters affecting the adsorption process. The model was developed based on artificial neural network considering a combination of linear and non-linear transfer functions which were designed inside the hidden layers. The optimum designed neural network indicated high accuracy in terms of fitting the data with R^2^ more than 0.99 which is a great agreement. The model was further assessed in prediction of adsorption capacity of the process and revealed that the initial solute concentration has major influence on the adsorption removal rate due to changing the mass transfer rate and driving force of the separation process. The modeling results showed that increasing the initial content of both heavy metal ions in the solution will increase the adsorption capacity (Qe) significantly. Moreover, it was confirmed that Cd had higher adsorption on the surface of MOF/LDH nanocomposite adsorbent which is due the strong interaction between the solute and the adsorbent surface. The ANN model strategy indicated to be rigorous and robust in simulation of adsorption data for the studied ions and can be further developed for other ions and organic materials.
